# Repair of a defect following the removal of an impacted maxillary canine by orthodontic tooth movement: a case report

**DOI:** 10.1186/1757-1626-3-62

**Published:** 2010-02-15

**Authors:** Wai Yip Lei, A Bakr M Rabie, Ricky WK Wong

**Affiliations:** 1 Discipline of Orthodontics, The University of Hong Kong, 2/F Prince Philip Dental Hospital, 34 Hospital Road, Sai Ying Pun, Hong Kong SAR, China

## Abstract

This case report describes a 13-year-old boy with alveolar bony defect resulted from surgical removal of impacted upper canine transposed in the anterior region. The boy had a normal occlusion with malposition of upper central and lateral incisors. The treatment objectives were to align teeth, close spaces by mesial movement of the buccal segments in the upper jaw to repair bone loss. Fixed appliance with palatal root torque was used for the mesial movements, levelling, and alignment of teeth.

Orthodontic tooth movement consisted of a sequence of root movement in a direction to increase the thickness of the labial cortical plate of bone, could ensure healthier periodontium. A healthier periodontium prior to space closure ensured repair of alveolar bony defect after surgical intervention. Orthodontic tooth movement should be added to our armamentarium for the repair of alveolar bony defect.

## Introduction

Surgical removal of impacted tooth in the anterior region can create an alveolar bone defect. Orthodontic tooth movement of adjacent teeth, which triggers bone remodelling, can be used to induce bone to repair the defect. However, this type of orthodontic movement is very challenging and it is related to a variety of problems. These problems and the strategies to overcome them are listed below.

Firstly, the condition requires substantial amount of bodily movement of adjacent teeth. The movement of force required to produce bodily movement is difficult to control [1]. Even when orthodontic forces are applied in the desired direction, it is difficult to produce the amount of root movement required because a large hyalinised layer will be created [2]. This will result in slow tooth movement and root resorption. The risk for the latter is increased if the tooth has short and blunted root [3]. In addition, the roots of the upper anterior teeth are close to the cortical bone of the maxilla, which is an area of reduced vascularisation. This results in delayed bone remodelling and tooth movement. In order to produce efficient root movement induce bone formation and reduce risk of root resorption, light orthodontic force will be needed. This can be achieved by segmental wire with frictionless mechanics coupled with slight activation during treatment.

On the other hand, the buccal bone covering the anterior teeth is thin. This creates another risk factor for bone dehiscence and gingival recession. To overcome this, palatal root torque is needed during orthodontic movement of the tooth to increase the buccal bone thickness, decrease the risk of bone dehiscence and decrease the risk of gingival recession [4,5].

To understand the above problem and to illustrate the technique and mechanics for the correction, it is desirable to use an actual case for presentation. Therefore, the following case with a bone defect coupled with short and blunt root of the adjacent teeth is presented.

## Case presentation

A 13-year-old boy was presented with a Class I skeletal base and a Class I incisor relationship with increased mandibular plane angle and increased lower facial heights. Extraoral examination demonstrated facial symmetry and a straight profile. The upper left central and lateral incisors were in crossbite. The upper dental centreline was deviated 1.0 mm to the left of the mid-facial axis. The lips were competent at rest. There was 3.0 mm of incisor show on smiling.

**Figure 1:**
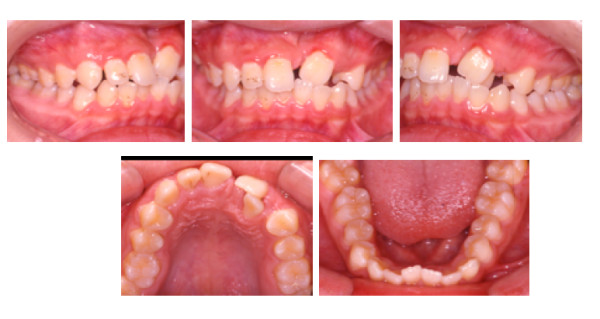
**Pre-treatment intraoral views illustrate the dimension of the alveolar bony defect located mesial to apical region of upper left central incisor**. The buccal view illustrates the relationship of the upper left central and lateral incisors.

Intra-oral examination revealed fair oral hygiene. There were no active carious lesions. In the maxillary arch, there was a bony defect mesial to the upper left central incisor. A space of 5.5 mm existed in the alveolar ridge where the upper left canine was extracted. The upper left central incisor was buccally displaced and the lateral incisor was palatally displaced. There was anterior mandibular displacement of 2 mm upon closure. The left central and lateral incisors were in crossbite and were retroclined relative to the maxillary plane (Figure 1).

In the mandibular arch the labial segment was mildly crowded and retroclined relative to the mandibular plane. There was a curve of Spee measuring 2 mm at its deepest part (Figure 1).

In centric occlusion, the incisor relationship was Class I with overjet of 4 mm at the right central incisors, and with a reverse overjet of 0.5 mm and a reduced and incomplete overbite. The molar relationship was Class I. There was a decreased buccal overjet at the posterior segments.

**Figure 2:**
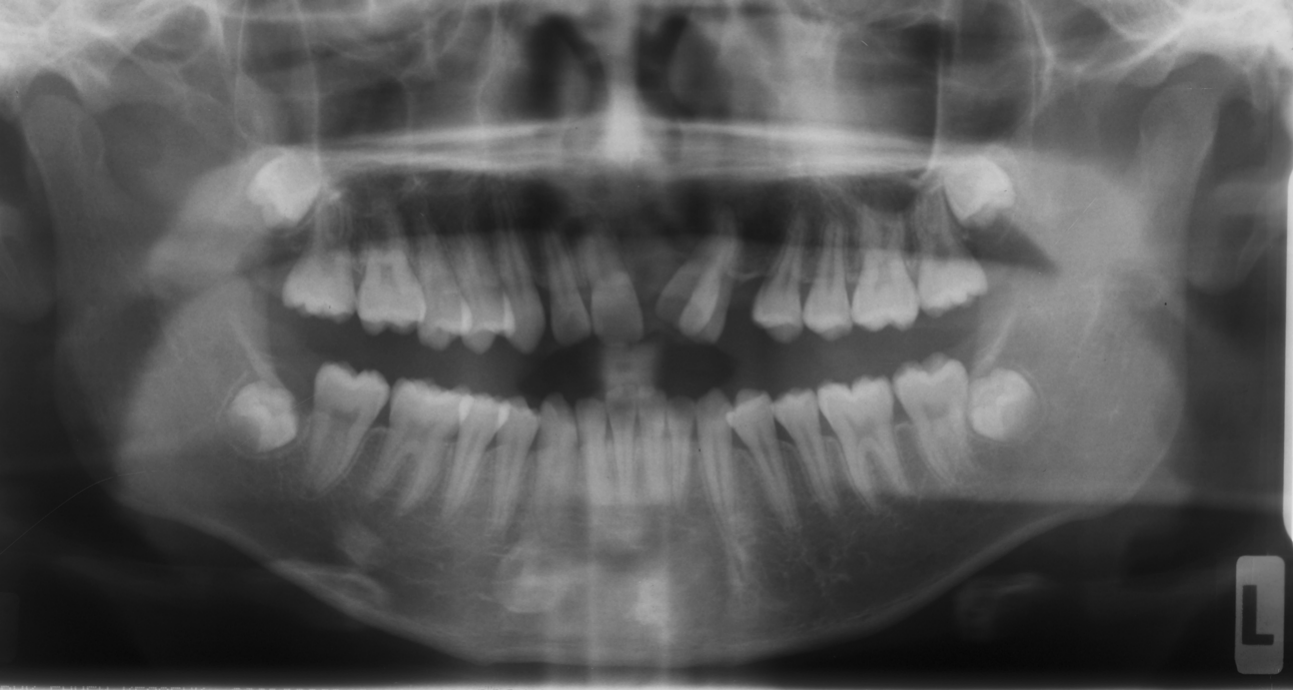
**Pre-treatment panoramic radiograph**.

Radiographic examination confirmed the absence of the upper right canine and the presence of unerupted third molars and no carious lesions were detected. The upper left central incisor showed short root, and the lower incisors showed pipette shaped roots (Figure 2).

**Figure 3:**
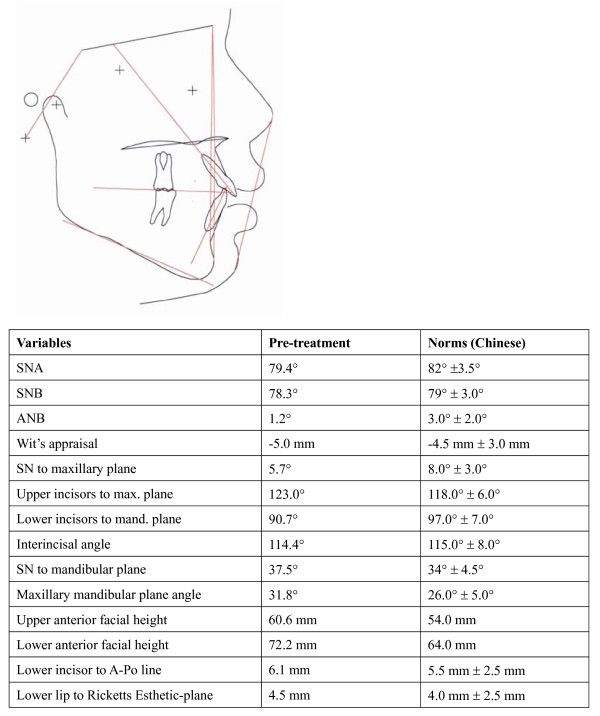
**Pre-treatment cephalometric tracing and analysis**. Cephalometric standards of southern Chinese [11].

The cephalometric analysis showed a skeletal Class I base (ANB and Wit's appraisal) with slightly retrognathic maxilla. The upper and lower facial heights were increased. There was an increased mandibular plane angle which was in agreement with increased lower facial height, but in good proportion with the upper facial height. The lower incisors were retroclined, and were on the Point A- Po line (Figure 3)

The aims of the treatment were: 1. To close upper anterior space by mesialization of 21, 2. To eliminate anterior crossbite by proclination of displaced upper anterior teeth, 3. To relieve lower crowding and flatten curve of Spee.

The treatment plan was orthodontic treatment with fixed pre-adjusted appliance without extraction in the upper arch; and extraction of 34 in the lower arch. The abnormal positioning of 21 and 22 posed the main problem, and was exaggerated by the bony defect. Proper biomechanics were needed to mesialize the displaced tooth 21 into the anterior space to repair the bony defect.

## Treatment progress

**Figure 4:**
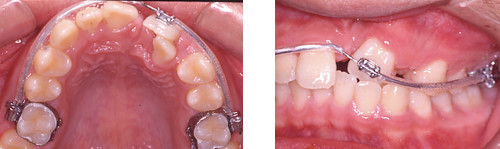
**Treatment Progress**. 0.017" × 0.025" TMA archwire, palatal root torque at tooth 21.

There was a thin labial plate of bone overlying the upper left central incisor. The treatment started with banding the upper molars, and the application of palatal root torque at tooth 21 on 0.017" × 0.025" TMA archwire (Titanium molybdenum alloy, Ormco Corporation, Glendora, USA). Application of palatal root torque would increase the thickness of bone at the labial aspect of the incisor (Figure 4). This would reduce the chance of gingival recession as the tooth was brought down to the occlusion. The long span of activation was aimed to maintain a light orthodontic force by increasing flexibility.

**Figure 5:**
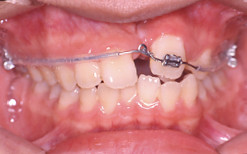
**Treatment Progress**. 0.017" × 0.025" TMA archwire with loop mesial to the 21, steel ligature to 21 to control the crown position, mesialize the root 21.

Seven months into treatment, there was remarkable increase in the bone at the buccal aspect of tooth 21. A loop was placed on the archwire mesial to tooth 21, and steel ligature was tied to the crown in order to control the crown position while uprighting the root mesially (Figure 5).

**Figure 6:**
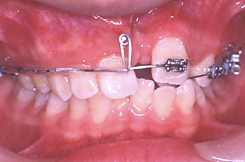
**Treatment Progress**. Closing loop on 0.017" × 0.025" TMA archwire to mesialize 21. Placement of fixed appliance on the upper left teeth, 0.016" NiTi archwire to align the teeth.

Nine months into treatment, a closing loop was placed to close the median diastema. There were four functions of this mechanics: 1. To close space; 2. To apply palatal root torque continuously; 3. To upright the central incisor; and 4. To provide a slight and long span of force application. This long span of force application would mean a light and continuous force being applied to the tooth. The alignment of upper left segment was also started with 0.016" nickel titanium (NiTi) archwire (Figure 6).

**Figure 7:**
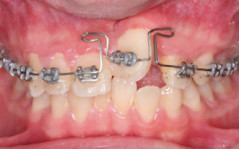
**Treatment Progress**. Butterfly loop on 0.017" × 0.025" TMA, extrude, palatal root torque application, derotate the tooth 21.

Fourteen months into treatment, fixed orthodontic appliance was placed in the whole upper arch. A butterfly loop was placed at the tooth 21 (Figure 7). The purposes were to extrude the tooth to occlusal level, to derotate the tooth and to continue to apply the palatal root torque. The lower arch was band and bonded with fixed orthodontic appliances at this stage.

**Figure 8:**
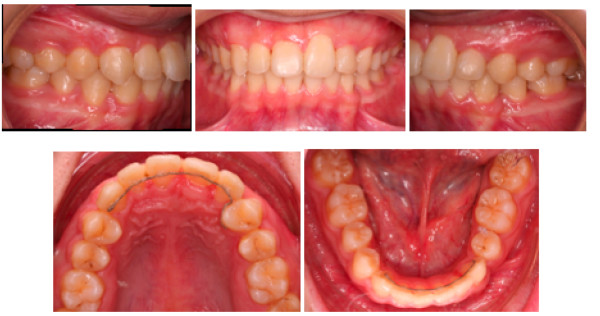
**Post treatment extraoral and intraoral photos**.

**Figure 9:**
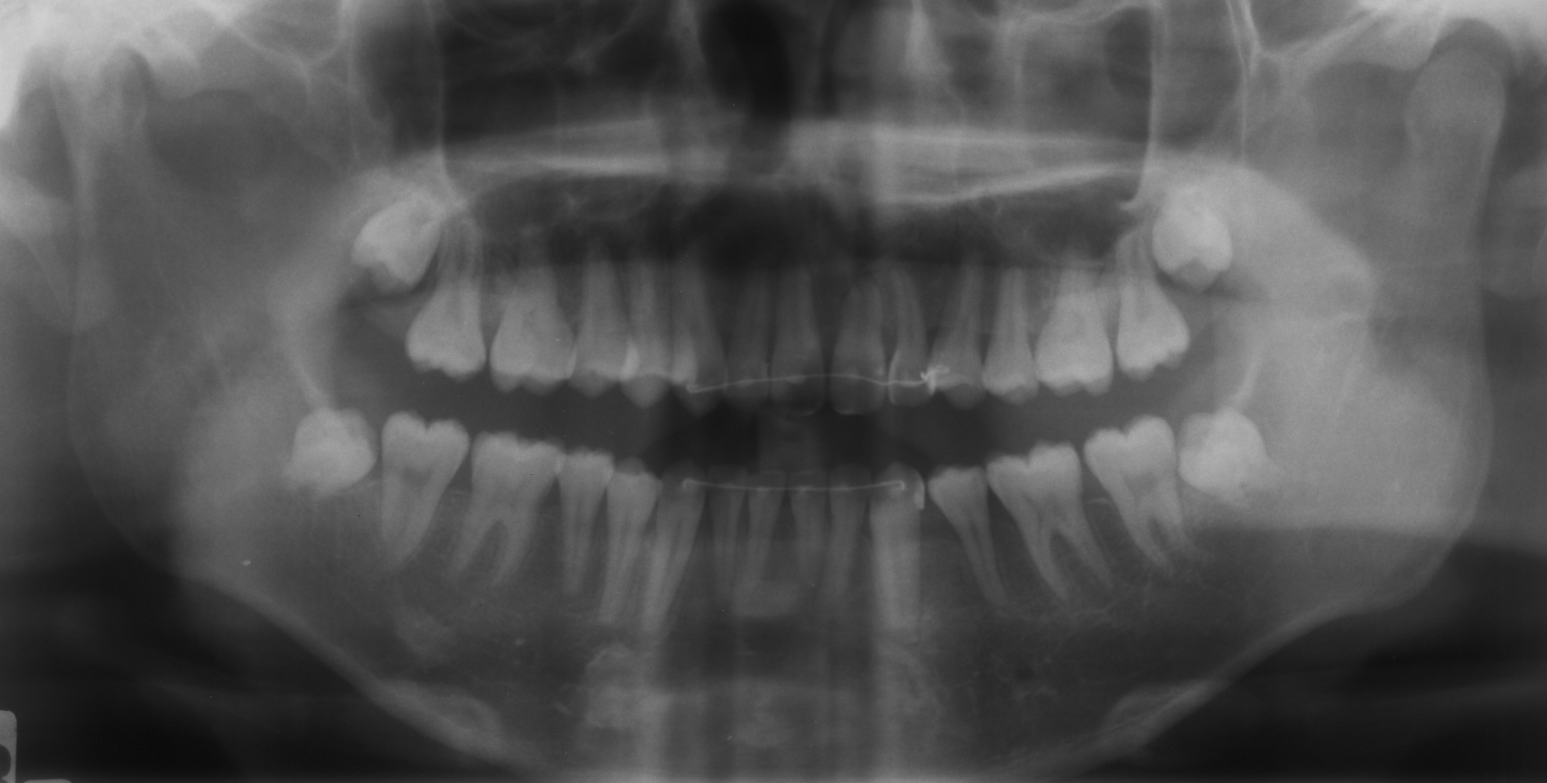
**Post treatment panoramic radiograph**.

The treatment was completed in 32 months. All objectives of treatment had been achieved (Figure 8 and 9). Buccal segment and incisor relationships were in Class I. Teeth were well aligned with good occlusal interdigitation.

## Discussion

Bone defect is one of the greatest problems in clinical orthodontics. Without bone, orthodontic tooth movement is not possible. Management of bony defect by bone grafting is the preferred method of treatment [6]. Bone grafting followed by subsequent orthodontic treatment has been shown to be highly successful in eliminating bony defect [7]. Orthodontic tooth movement is a stimulating factor for bone apposition [8]. It was also shown that enhanced bone healing following orthodontic movement where the defect involved periodontal structures [9]. Total bony apposition was 6.5 fold larger with the orthodontic tooth movement into the surgical bony defects in rats [8]. It has been reported that slight gentle orthodontic forces from the use of laceback ligature technique is effective in correction of bone deficient alveolar ridge [10].

This case report demonstrated an alternative method in the correction of bony defect. The treatment sequence and mechanics took into consideration the advantages of frictionless mechanics (closing loop), and the light force exerted by sectional wire on a long span archwire. These measures were important in addressing the concern of this case report where anterior teeth with short and blunt roots needed to be moved bodily over a considerable distance to close the median diastema region. With proper biomechanics to move the root slowly towards the centreline; this allowed the bone to remodel. The bone defect was narrowed down by bone deposition around the root during the gradual space closure period.

In addition, emphasis was given on the importance of application of palatal root torque. Palatal root torque was applied over a considerable period of time. This increased the labial bone thickness, which in turn increased the width of the attached gingival. These measures prevented gingival recession which would occur if there was insufficient bone labial to the anterior teeth. Move the teeth away from cortical plate subsequently increased vascularisation, increase orthodontic tooth movement, and reduce the risk of root resorption. Maintaining controlled light gentle orthodontic force provided extra care for the orthodontic movement of anterior teeth with short roots.

## Conclusions

Orthodontic tooth movement with wire sequence and mechanics of moving the root in a direction to increase the thickness of the labial plate of bone can ensure healthier periodontium. Healthier periodontium prior to space closure promotes repair of alveolar bony defect after surgical intervention. Orthodontic tooth movement should be added to our armamentarium for the repair of alveolar bony defect.

## Consent

Written informed consent was obtained from the patient for publication of this case report and accompanying images. A copy of the written consent is available for review by the Editor-in-Chief of this journal.

## Competing interests

The authors declare that they have no competing interests.

## Authors' contributions

WYL conducted the treatment and was a major contributor in writing the manuscript. ABMR was the supervisor of the case. RWKW contributed in writing the manuscript. All authors read and approved the final manuscript.
